# Large Distortion of Fused Aromatics on Dielectric Interlayers Quantified by Photoemission Orbital Tomography

**DOI:** 10.1021/acsnano.2c08631

**Published:** 2022-10-14

**Authors:** Philipp Hurdax, Christian S. Kern, Thomas Georg Boné, Anja Haags, Michael Hollerer, Larissa Egger, Xiaosheng Yang, Hans Kirschner, Alexander Gottwald, Mathias Richter, François C. Bocquet, Serguei Soubatch, Georg Koller, Frank Stefan Tautz, Martin Sterrer, Peter Puschnig, Michael G. Ramsey

**Affiliations:** † Institute of Physics, University of Graz, NAWI Graz, Universitätsplatz 5, 8010Graz, Austria; ‡ Peter Grünberg Institute (PGI-3), Forschungszentrum Jülich, 52425Jülich, Germany; § Jülich Aachen Research Alliance (JARA), Fundamentals of Future Information Technology, 52425Jülich, Germany; ∥ Experimentalphysik IV A, RWTH Aachen University, 52074Aachen, Germany; ⊥ Physikalisch-Technische Bundesanstalt (PTB), 10587Berlin, Germany

**Keywords:** molecular distortion, dielectric thin films, photoemission orbital tomography, perylene-3,4,9,10-tetracarboxylic dianhydride, density functional theory

## Abstract

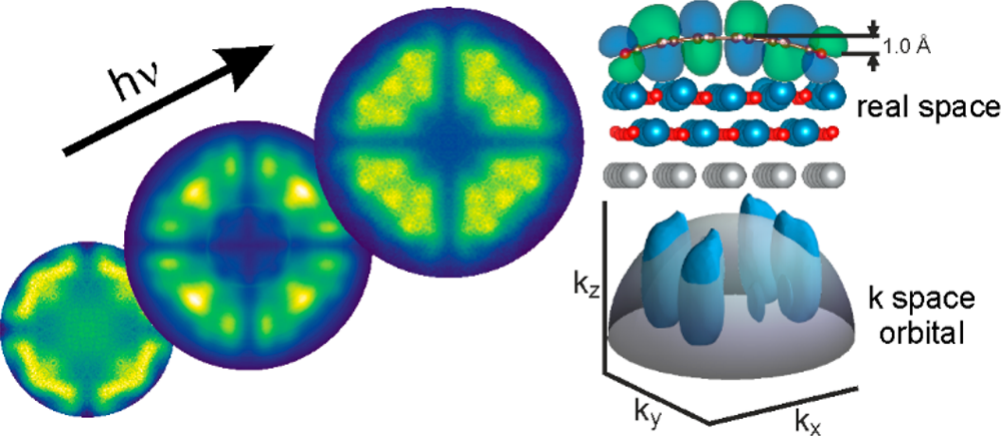

Polycyclic aromatic compounds with fused benzene rings offer an extraordinary versatility as next-generation organic semiconducting materials for nanoelectronics and optoelectronics due to their tunable characteristics, including charge-carrier mobility and optical absorption. Nonplanarity can be an additional parameter to customize their electronic and optical properties without changing the aromatic core. In this work, we report a combined experimental and theoretical study in which we directly observe large, geometry-induced modifications in the frontier orbitals of a prototypical dye molecule when adsorbed on an atomically thin dielectric interlayer on a metallic substrate. Experimentally, we employ angle-resolved photoemission experiments, interpreted in the framework of the photoemission orbital tomography technique. We demonstrate its sensitivity to detect geometrical bends in adsorbed molecules and highlight the role of the photon energy used in experiment for detecting such geometrical distortions. Theoretically, we conduct density functional calculations to determine the geometric and electronic structure of the adsorbed molecule and simulate the photoemission angular distribution patterns. While we found an overall good agreement between experimental and theoretical data, our results also unveil limitations in current van der Waals corrected density functional approaches for such organic/dielectric interfaces. Hence, photoemission orbital tomography provides a vital experimental benchmark for such systems. By comparison with the state of the same molecule on a metallic substrate, we also offer an explanation why the adsorption on the dielectric induces such large bends in the molecule.

Fused aromatic molecules are shaped by sp^2^ hybridization, which results in a planar network of bonds and an electronic structure characterized by a series of σ-orbitals and π-orbitals, the latter having their electron densities on either side of the molecular plane. This electronic configuration results in a stiffness of carbon skeletons ranging from small molecules such as benzene over to one or two-dimensional structures such as linear acenes or coronene to graphene. Thus, those structures are comparably resistant to geometric distortions, which actually may be desirable, as distortion has profound effects on the optoelectronic^1−8^ and chemical properties^9−12^ of various materials. Bonding on metal surfaces is expected to soften the carbon backbone through hybridization, thereby allowing for bonds, e.g., with functional groups, to induce geometric distortions. Indeed, molecular distortions have been observed on metal surfaces, such as a bend of perylene-3,4,9,10-tetracarboxylic dianhydride (PTCDA) detected by X-ray standing waves (XSW)^13^ or a corrugation of graphene detected by atomic force microscopy (AFM).^14^ However, in these cases, the out-of-plane distortion of the carbon backbone is small (≤0.1 Å). Here, we report a much larger distortion of a π-conjugated planar molecule. Moreover, we observe it on a dielectric thin film, where there is no hybridization between the substrate states and the π-system of the carbon core of the molecule.

Geometric distortions can be inferred by several methods, such as dynamic low-energy electron diffraction, X-ray photoelectron diffraction, and XSW. These, however, pose strict requirements to the systems investigated. For instance, the XSW technique has been used successfully to detect adsorption heights of constituent atoms,^15−23^ but it requires atomic species to be energetically distinguishable in X-ray photoelectron spectroscopy (XPS) and therefore cannot discern between carbon atoms with the same local chemical environment.

The photoemission orbital tomography (POT) technique essentially produces images of molecular orbitals in momentum space (k-space), which are related to the real space orbitals by the square of their Fourier transform.^24,25^ Since orbitals are a direct result of the details of the internal atomic structure, POT should be able to detect conformational changes on adsorption directly. Indeed, POT has been demonstrated to shed light on subtle questions such as the degree of aromaticity in kekulene.^26^ POT has also been used successfully to detect and quantify geometric changes of *p*-sexiphenyl, namely the planarization of the molecule upon adsorption on metals and oxide thin films, when there is charge transfer to the molecule, thereby removing the torsional angle between the phenyl rings around the nominal single bonds of the molecule.^24,27,28^ In this work, we show that POT can be used to detect and quantify even more subtle geometric changes, namely the bend of the molecule PTCDA.

PTCDA has been studied on various substrates as a model molecule. POT of PTCDA has been conducted on metals such as Ag(001),^29^ Ag(110),^30−34^ Ag(111),^35,36^ Cu(100),^37^ and oxidized Cu(100).^38,39^ In all these cases, the patterns of the photoemission distribution from the frontier orbitals were in close agreement with the patterns calculated for flat, oriented molecules in the gas phase (approximating the final state of photoemission by a plane wave)^40^ and showed no photon energy dependence.^32,41^

On oxide films, deviations of the orbitals on adsorption might be deemed even more unlikely, since the dielectric interlayer decouples the molecular wave function from the metallic wave function, thereby preventing any hybridization.^42^ Indeed, POT momentum maps of the frontier orbitals of both pentacene^43^ and *p*-sexiphenyl^28^ on epitaxial MgO(001) films on Ag(001) were in very good agreement with gas-phase simulations of planar molecules.

Here, we show that for PTCDA on MgO(001)/Ag(001), there are large differences between the experimental momentum maps of the frontier orbitals and theoretical ones simulated for the gas phase. Most notably, for this system, the momentum maps display a very strong photon energy dependence. With the support of density functional theory (DFT) calculations, this is identified as the result of a significant bend in the molecular backbone arising on adsorption. By contrasting the situation to adsorption on Ag(001), with the same adsorption configuration, we can understand the bend on MgO as arising from the higher electronic hardness of the dielectric film compared to the metal surface.

## Results and Discussion

**Figure 1:**
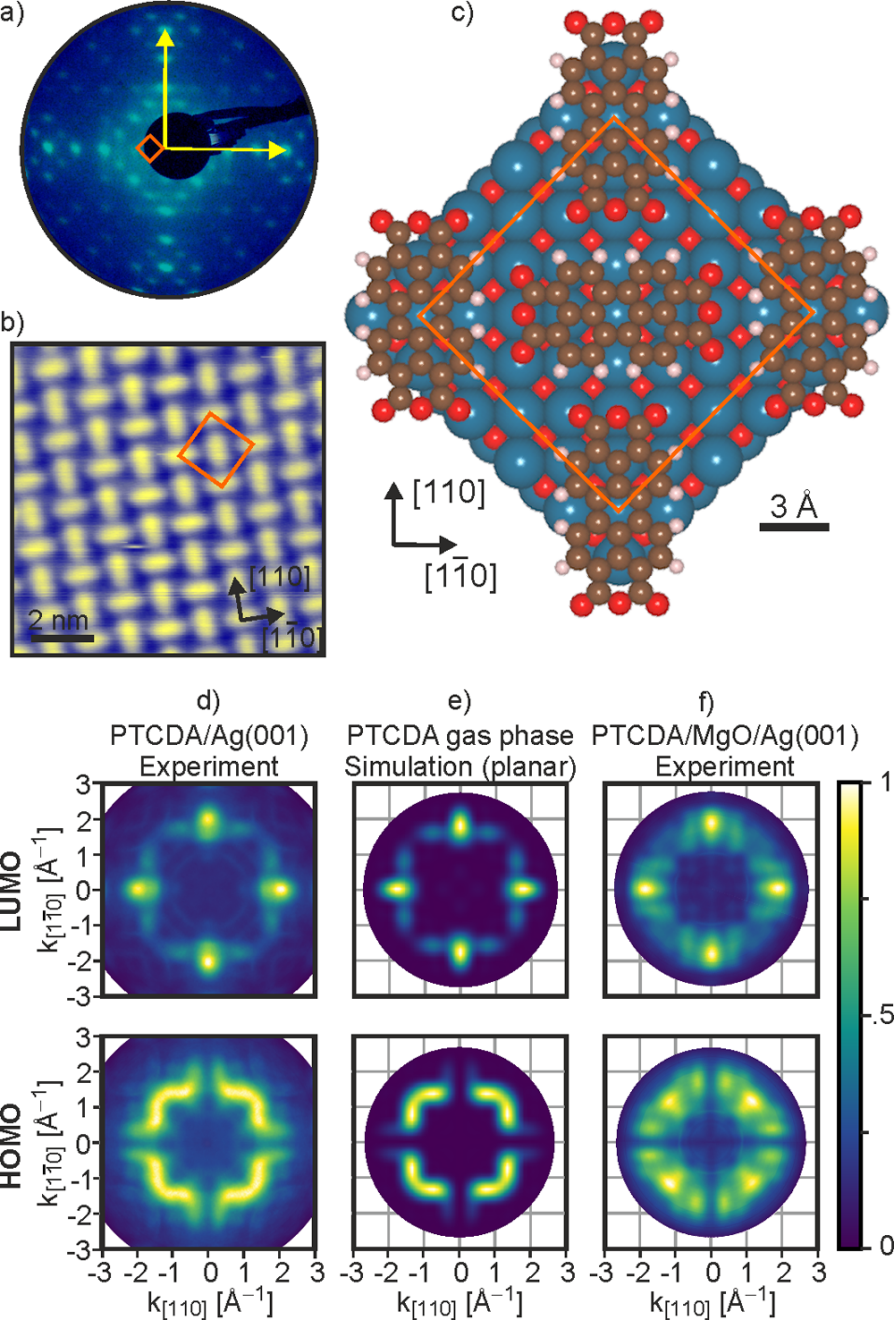
Identical structure but different momentum maps of PTCDA on Ag(001) and on MgO(001)/Ag(001). (a) LEED pattern of the PTCDA monolayer on 2 ML MgO(001)/Ag(001) at an energy of 50 eV. The substrate unit cell vectors are indicated by yellow arrows, and the unit cell of the PTCDA overlayer is shown as an orange square. (b) Structure of the PTCDA monolayer measured by STM (*V*_bias_ = 1.35 V, *I*_t_ = 61 pA). (c) Schematic of the adsorption geometry. (d) Experimental momentum maps of the LUMO and the HOMO of PTCDA/Ag(001) measured at a photon energy of 57 eV. (e) Simulated maps for LUMO and HOMO of gas-phase PTCDA at a photon energy of 35 eV with two orthogonal orientations. (f) Experimental momentum maps of LUMO and HOMO of PTCDA/MgO(001)/Ag(001) measured at a photon energy of 35 eV. The binding energies of the measured LUMO and HOMO with respect to the Fermi level are, respectively, 0.5 and 1.7 eV in (d) and 1.6 and 2.8 eV in (f).

MgO forms well-ordered (001)-oriented films on Ag(001) due to the close match of their lattices.^44,45^ Adsorbed monolayers (MLs) of PTCDA on MgO(001)/Ag(001) are identical to PTCDA on Ag(001) in terms of the superstructure. This is evident in the low-energy electron diffraction (LEED) image in Figure 1a, which reveals a PTCDA superlattice with the epitaxial matrix:
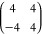


The scanning tunneling microscopy (STM) image in Figure 1b confirms this and shows the orientation of the long molecular axes aligned alternatingly along the two principal crystallographic directions, [110] and[11̅0], of the substrate. Thus, PTCDA adopts the same structure on MgO(001)/Ag(001) as on Ag(001),^13^ as illustrated in Figure 1c.

Despite this close similarity, the POT momentum maps of the frontier molecular orbitals are different on the two substrates. Figure 1d shows momentum maps of the PTCDA lowest unoccupied and highest occupied molecular orbitals (LUMO and HOMO), respectively, on Ag(001) measured at a photon energy of 57 eV. Significantly, their appearance is in close agreement with momentum maps measured at lower photon energy^29^ and with that of the maps simulated for two gas-phase planar molecules oriented 90° with respect to each other shown in Figure 1e. In contrast, the measured momentum maps of PTCDA on MgO(001)/Ag(001) displayed in Figure 1f, although recognizable from their nodal structures,^38^ are strikingly different from the gas-phase maps simulated for the same photon energy (Figure 1e). Of note, for the LUMO, which is occupied by tunneling from the underlying metal, is the elongation of the minor lobes and the appearance of emissions at (±0.5, ±0.5) Å^–1^. For the HOMO the distinct “W” shape in each quadrant is replaced by a more diffuse emission pattern containing a number of maxima, the most intense one located at (±1.2, ±1.2) Å^–1^. We formulate the hypothesis that these significant changes of the emission patterns of PTCDA adsorbed on MgO are caused by a distortion of the molecular orbitals on adsorption due to a significant bend of the backbone of PTCDA.

**Figure 2:**
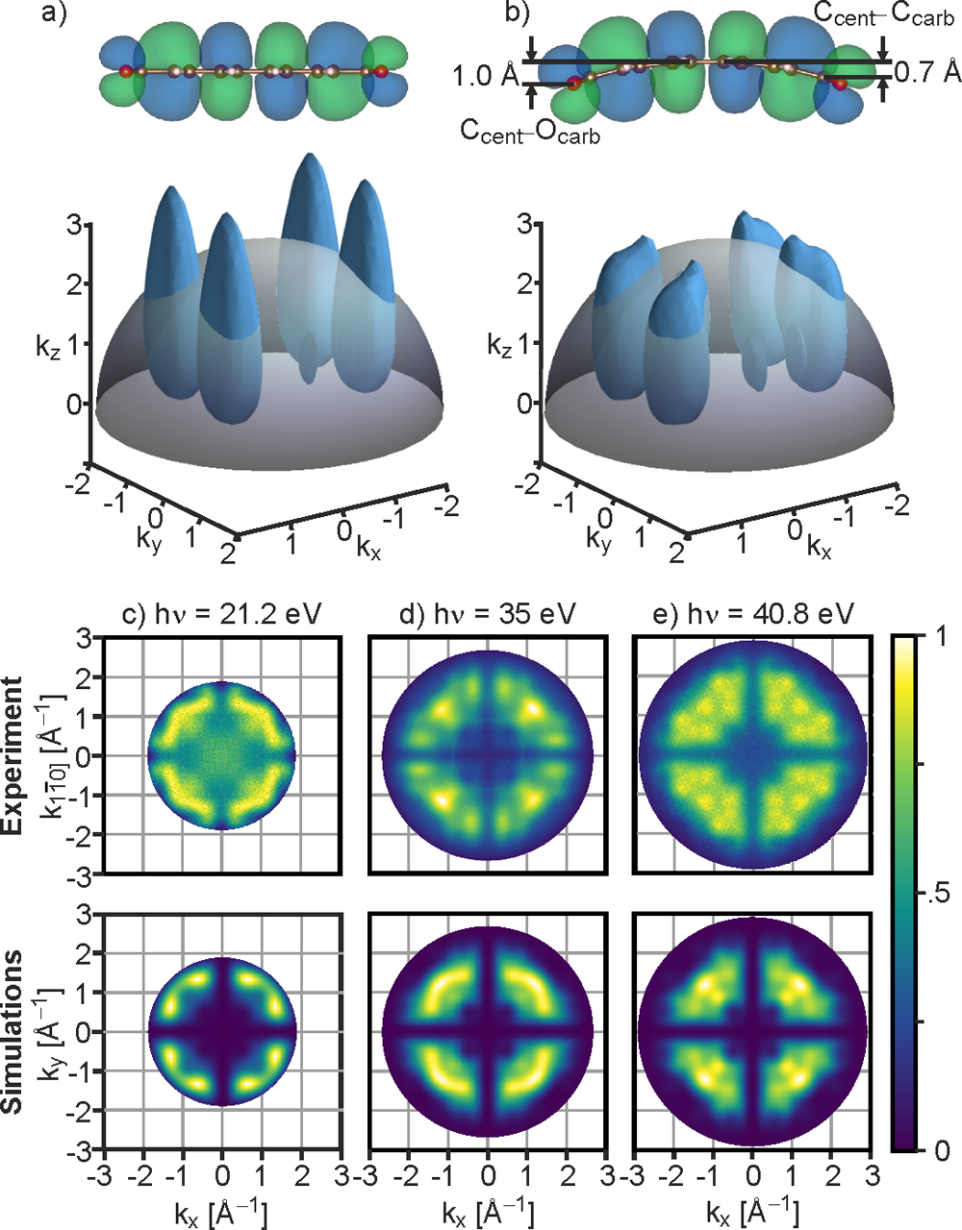
Momentum maps of PTCDA on MgO(001)/Ag(001) at different photon energies. (a, b) Side view in real space and corresponding three-dimensional k-space density (square of the Fourier transform of the real space orbital) of the HOMO of the PTCDA molecule in its planar geometry (a) and in the bent geometry (b) as obtained by on-surface DFT calculations. The bend across the carbon backbone (0.7 Å) and the total bend of the molecule (1.0 Å), including the end oxygens, are indicated. The gray hemispheres represent the Ewald sphere for a photon energy of 35 eV. (c–e) Symmetrized experimental momentum maps (upper panel) of the PTCDA HOMO on MgO(001)/Ag(001) taken at photon energies of (c) 21.2 eV, (d) 35 eV, and (e) 40.8 eV, and (lower panel) simulated momentum maps of the HOMO for isolated molecules in the bent geometry at the experimental photon energies.

The presence of a bend can already be inferred, even without involved DFT calculations of the PTCDA/MgO(001)/Ag(001) heterostructure, by considering momentum maps at different photon energies. This is illustrated in Figure 2 for a planar (Figure 2a) and bent (Figure 2b) PTCDA molecule with the example of its HOMO. Momentum maps can be viewed as spherical cuts through the square of the orbitals in k-space projected, in the case of a flat-lying molecule, onto the molecular plane.^24^ The radius of the sphere, representing the Ewald sphere of photoemission, increases with the square root of the photon energy. Changing the photon energy therefore entails a change in the vertical component of the momentum (*k*_*z*_) across the map. Planar π-systems as the PTCDA HOMO shown in Figure 2a have practically no photon energy dependence of the (*k*_*x*_, *k*_*y*_) position of the main emissions, because the periodicities of the wave function in the *x*,*y* plane do not depend on the *z*-coordinate. In other words, their wave function can be approximated by the product of a part depending on the in-plane coordinates and a part depending on the vertical coordinate *z*. In momentum space (Figure 2a), this leads to lobes which are oriented perpendicular to the molecular plane. Thus, as the Ewald sphere expands with increasing photon energy, it will always intersect the orbital at the same (*k*_*x*_, *k*_*y*_) corresponding to a particular periodicity of the wave function. Experimentally, this has indeed been observed for PTCDA monolayers on the three facial Ag surfaces as well as on bare and oxidized Cu(100).^32^ However, if the molecule adopts a nonplanar geometry, such as the bent one shown in Figure 2b, the orbitals must be slightly deformed due to local distortions of atomic orbitals constituting the molecular π-system. Thus, *k*_*z*_-dependent substructures are introduced in the lobes of the three-dimensional k-space orbital (Figure 2b), which will result in a strong photon energy dependence of the momentum maps.

To investigate whether the emission patterns in the momentum maps of PTCDA/MgO(001)/Ag(001) are photon energy dependent, additional momentum maps were measured with a lab-based instrument using unpolarized He I and He II radiation of 21.2 and 40.8 eV, respectively. In Figure 2c–e (upper panel), the symmetrized experimental maps of the HOMO are shown together with the momentum map recorded at 35 eV with p-polarization obtained with synchrotron radiation. At low photon energy (*h*ν = 21.2 eV, Figure 2c), the measured momentum map is almost identical to the simulated map of the gas-phase planar PTCDA molecule shown in Figure 1e. This is in contrast to the map recorded at 35 eV shown in Figure 2d, which exhibits notable differences as already mentioned. Further significant changes in the number of emission maxima can be seen when increasing *h*ν from 35 to 40.8 eV (Figure 2e). Note that the map of the LUMO, which is available in the Supporting Information (Figure S1), also undergoes clear changes with photon energy. We can thus conclude that a photon energy-dependent study of PTCDA momentum maps on MgO(001)/Ag(001) with POT indeed supports the hypothesis of a nonplanar molecular geometry.

DFT calculations of PTCDA on MgO(001)/Ag(001) also suggest a significant bend of the molecule. In order to separate the effect of the bend on the momentum maps from a possible influence of the environment at the adsorption site, we first calculate the orbitals for an isolated gas-phase molecule but in the geometry obtained from the calculation on the surface. Also note that in the simulation, the structure of the molecular layer is taken into account by superimposing momentum maps of two perpendicularly oriented PTCDA molecules. The resulting HOMO momentum maps simulated for the same photon energies as used in the experiment are shown in Figure 2c–e (lower panel, see Supporting Information SI2, Figure S2, for the photon energy dependence of the simulated LUMO maps). At a low photon energy of 21.2 eV (Figure 2c), like the experimental map, the simulated map resembles that of the planar molecule. As the photon energy rises (Figure 2d,e), the simulated maps show clear changes, which qualitatively reproduce the trend seen in the experimental maps. However, the quantitative agreement is not perfect. We notice that systematically a higher photon energy in the simulations would be required to achieve a better match with the experimental maps. The theoretical map at a photon energy of 35 eV is in closest agreement to the experimental map at 21.2 eV, and the theoretical map at 40.8 eV resembles the experimental map at 35 eV. This discrepancy between theoretical and experimental maps, also seen in the more subtle changes of the LUMO (see Supporting Information SI1, Figure S1), might be the consequence of a bend larger than this particular DFT calculation would suggest.

**Figure 3:**
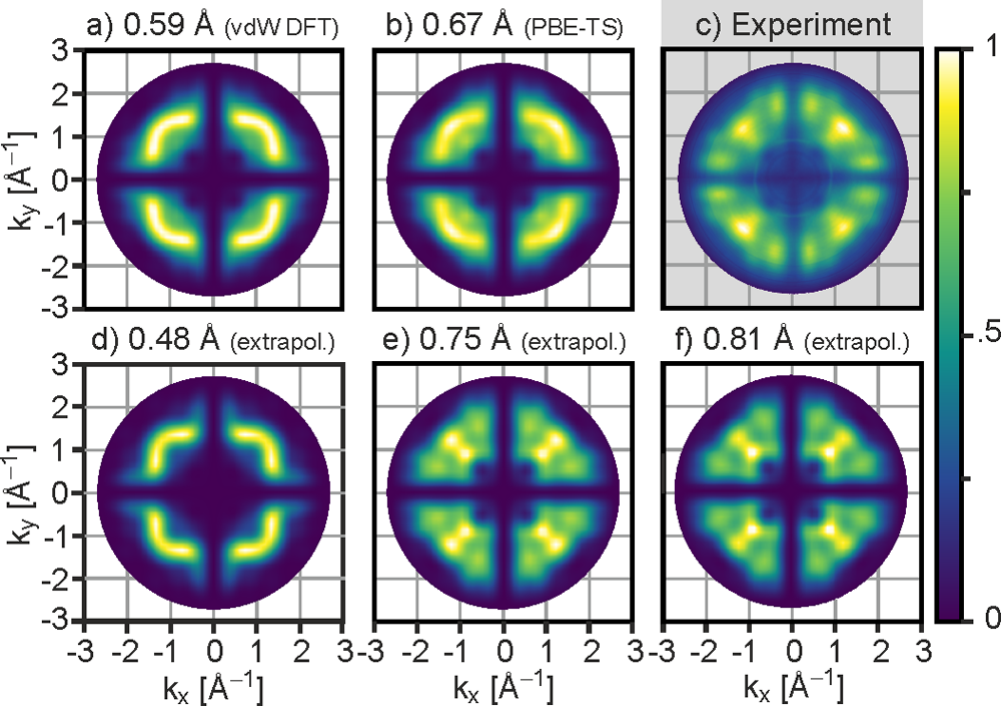
Dependence of the PTCDA HOMO momentum map on the bend. (a, b) Simulated momentum maps of isolated PTCDA with the geometry obtained by on-surface calculations using the van der Waals correction schemes vdW-DFT and PBE-TS, respectively. (c) Experimental momentum map of PTCDA on MgO(001)/Ag(001). (d–f) Simulated momentum maps of isolated PTCDA for bends extrapolated from the vdW-DFT geometries (see Supporting Information SI3). All simulated maps are shown at an energy corresponding to the experimental photon energy of 35 eV and for two orthogonal orientations of the molecule. The bends, C_cent_–C_carb_ height differences, are listed on top of the images.

To test if a different bend of the molecule can account for the remaining mismatch between theory and experiment and to gain a better understanding of the effect of the bend on the emission patterns, additional DFT calculations for the PTCDA/MgO(001)/Ag(001) heterostructure have been carried out. These were performed for perfectly stoichiometric MgO interlayers and MgO layers for which oxygen was introduced at the MgO/Ag interface in order to account for a realistic range of work functions.^46−48^ Moreover, as it is yet unclear which theoretical methodology is best suited for the complex dielectric/metal substrate system, two different van der Waals correction schemes have been employed: the Tkatchenko–Scheffler method^49^ on top of Perdew–Burke–Ernzerhof DFT methodology (PBE-TS) and a van der Waals DFT functional (vdW-DFT).^50,51^ To quantify the degree of bending, we take the height difference between the central carbon atoms of the molecule (C_cent_) and the carbon atoms of the carboxyl groups (C_carb_), as illustrated in Figure 2b. Using this definition, the calculated bends are found to vary by 0.05 Å depending on the work function of the substrate (which controls the degree of charge transfer into the LUMO) and by 0.1 Å depending on the van der Waals correction scheme applied (see Supporting Information SI3, Table S1). On a stochiometric MgO film, PBE-TS yields a bend of 0.67 Å, while vdW-DFT leads to a bend of only 0.59 Å. Based on these geometries, we have simulated maps of the HOMO for *h*ν = 35 eV using the same procedure as described above, see Figure 3a,b. Although the larger bend obtained with PBE-TS yields a slightly better agreement, neither can be considered to well reproduce the experimental map shown in Figure 3c. Thus, we have also simulated momentum maps for a larger range of bends by extrapolating to smaller and larger values, respectively. When the bend is reduced to 0.48 Å (Figure 3d), the momentum map adopts the distinct W pattern in each of the four quadrants characteristic for the planar molecule (cf. Figure 1e). Conversely, increasing the bend slightly to 0.75 Å (Figure 3e) leads to further significant changes: The former W shaped pattern has broken up into four separate maxima, more similar to the experiment. A further increase in the bend to 0.81 Å (Figure 3f) causes the simulation to diverge from the experiment: The region of high intensity 45° to the principal azimuth becomes sharper and its dominant maximum changes from the experimental (±1.2, ±1.2) Å^–1^ to (±0.9, ±0.9) Å^–1^. Therefore, a bend of 0.81 Å overshoots the experimental momentum map and the best match is achieved at a bend around 0.75 Å.

**Figure 4:**
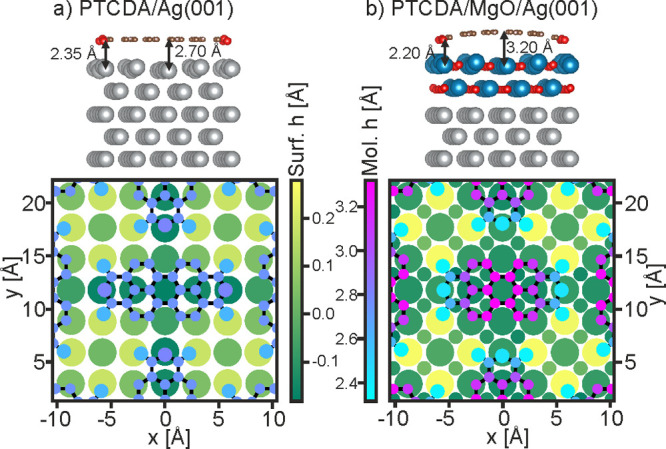
Comparing the atomic positions of PTCDA on Ag(001) and MgO(001)/Ag(001). Side view and top view of PTCDA on Ag(001) (a) and on 2 ML MgO(001)/Ag(001) (b). Both structures have been obtained from PBE-TS DFT calculations. In the side views, the O atoms are shown in red, the C atoms in bronze, the Ag atoms in silver, and the Mg atoms in blue. In the top views, atoms of both the substrate surface (Surf. h) and the molecule (Mol. h) are color-coded according to their elevations relative to the average height of the top substrate layer (atoms from smallest to largest are C, O, Ag, Mg).

The fact that the momentum map at a bend of 0.48 Å is hardly distinguishable from the map of the planar molecule suggests that the effect of the bend on photoemission patterns is nonlinear and the method only becomes highly sensitive above a critical threshold of the distortion. This also partly explains why the deviations from the planar conformation have not been observed in POT on any metal despite the presence of a small bend.^13^ Moreover, it needs to be considered that while on MgO the molecular bend extends across the entire molecular backbone, on Ag(001), as on other Ag surfaces, most of the height difference arises from the oxygens bending toward the substrate with the central carbon core of the molecule remaining essentially flat (see side view in Figure 4a).^13,52^ The maximum atomic height difference among carbon atoms amounts to only 0.1 Å for PTCDA on Ag(001) according to X-ray standing wave measurements and dispersion-corrected DFT.^13^ This is shown in the top view of Figure 4a, where atoms are colored according to their calculated heights. A bend restricted to the oxygens at the edges of the molecule leaves the frontier π orbitals mostly unaffected, since the latter are located predominantly in the region of the carbon backbone. In contrast, on MgO(001)/Ag(001), the bend is calculated to extend over the whole range of the molecule, as shown in Figure 4b.

To test if the discrepancy between experimental and simulated momentum maps might be explained by factors other than an altered shape of the molecular backbone, additional calculations have been carried out. These comprised (i) a more accurate description of the final state with time-dependent DFT (TD-DFT), (ii) accounting for intermolecular interactions in the free-standing monolayer as well as (iii) including the substrate. An overview of all these calculations can be found in the Supporting Information (SI4, Figures S3–S5). Of the three factors, point (iii) has the most significant effect on the momentum maps. However, none of them leads to a clear improvement in the agreement between theory and experiment. While the effects of these three factors impose an uncertainty on a quantification of the bend using POT, their investigation still confirms that a bend larger than suggested by DFT is required for theory to reproduce the experiment. Hence, we conclude that momentum maps are very sensitive to the magnitude of the bend and POT can thus serve as a benchmark to select the best computational approach for a given system.

To understand the origin of the strong bend of the molecular backbone when PTCDA adsorbs on MgO(001)/Ag(001), it is instructive to compare the situation to the one on Ag(001), where no significant bend occurs.^13^ As depicted in Figure 4, on both substrates the principle bonding is effectuated by the interaction of the carboxylic oxygens (O_carb_) at the corners of the molecule with the metal atoms underneath them. This leads to the latter being pulled above the substrate surface plane on both substrates, albeit to a greater extent for MgO, with O_carb_–Ag and O_carb_–Mg bond lengths of 2.35 and 2.2 Å, respectively. However, despite the carboxylic oxygens having similar heights above the substrate surface planes (∼2.5 Å), on Ag the carbon backbone is drawn close to the surface (2.7 Å) and remains essentially planar, while on MgO it bends with its center calculated to be 3.2 Å above the surface. This large height is similar to that obtained for a variety of aromatic molecules without functional anchor groups on MgO and can be attributed to the pushback effect of Pauli exclusion. While the quasi-free electrons of the Ag substrate can give way to create space for the electrons of the perylene core, the electrons of the dielectric are confined and cannot retract, thus preventing a closer approach of the molecular backbone. We suggest that the large bend of PTCDA on MgO/Ag expresses a more general aspect of adsorption on dielectrics, whose electronic hardness can lead to significantly stronger alterations of molecular geometries compared to metals.

## Conclusions

With the POT technique, we have shown that compared to metal surfaces, dielectric interlayers have the capacity to lead to greatly increased geometric distortions of adsorbed molecules containing anchor groups. POT can detect these structural modifications via the changes in the orbital structure of the molecules. As such, it has advantages over diffraction techniques whose requirement of long-range order can make them difficult to apply to heterogeneous systems such as the MgO/Ag substrate considered here. A noticeable photon energy dependence of the photoemission patterns of the frontier π-orbitals serves as a clear indication of a nonplanar adsorption geometry. In combination with simulations of the photoemission patterns, our experiments revealed the bend of the PTCDA backbone induced on adsorption on MgO(001)/Ag(001) to be larger than that predicted by DFT calculations with different methodologies. This suggests that POT can serve also as a benchmark for DFT.

We suggest that, as photoemission momentum maps reflect the orbital structure in k-space, when combined with DFT, the POT technique has the potential to determine the exact shape of nonplanar adsorbates by detailed studies of the photon energy dependence. Moreover, the sensitivity of POT to the bending of adsorbed molecules in conjunction with the possibility to combine POT with ultrafast time-resolved photoemission^39^ in principle provides the opportunity to study the nuclear dynamics, e.g., in surface chemical reactions, through its influence on molecular orbitals.

## Methods

All sample preparations and photoemission orbital tomography (POT) experiments were performed under ultrahigh-vacuum (UHV) conditions at a base pressure of about 3 × 10^–10^ mbar. A clean Ag(001) surface was obtained by cycles of Ar^+^ ion sputtering and annealing at 500 °C. MgO(001) films were grown by Mg evaporation in an oxygen environment (O_2_ pressure of 10^–6^ mbar) at a substrate temperature of 270 °C. Mg fluxes were of the order of 1 Å/min as calibrated by a quartz microbalance. After the growth of MgO, the sample was slowly (approximately 2.5 °C/min) cooled to room temperature (RT). All MgO films grown for this study had a nominal thickness of 2 ML. A monolayer of perylene-3,4,9,10-tetracarboxylic dianhydride (PTCDA) on 2 ML MgO(001)/Ag(001) was prepared by sublimation of solid PTCDA from a home-built evaporator with the substrate held at RT.

POT experiments were carried out at RT in two different UHV chambers. The experiments at a photon energy of 35 eV were conducted at the Metrology Light Source insertion device beamline of the Physikalisch-Technische Bundesanstalt^53^ using a toroidal electron analyzer.^54^ The sample was illuminated by p-polarized light at an angle of 40° to the surface normal. As momentum maps were acquired by recording the photoemission intensity in the incidence plane while rotating the sample around its normal, the polarization factor |**A**·**k**|^2^ depends only on the radial coordinate of the momentum maps.

For additional POT experiments at photon energies of 21.22 and 40.80 eV, we used a NanoESCA system by Scienta Omicron. The He I and II radiation from an unpolarized HIS 14 HD excitation source (Focus GmbH) was incident at an angle of 68° to the surface normal. Due to the focusing mirror, the sample was illuminated by 30.6% s-polarized and 69.4% p-polarized light. This results in a decrease of intensity in the lower half of the momentum maps due to the polarization factor |**A**·**k**|^2^ and a corresponding asymmetry of the momentum maps. In order to obtain a symmetric appearance more easily comparable to the k maps obtained with the toroidal electron analyzer, and to improve the signal-to-noise ratio, the data were subsequently symmetrized by mirroring the half of the map with more significant contributions of the orbitals to the intensity and averaging the map over instances rotated by 0°, 90°, 180°, and 270°.

In POT, the momentum map of photoemission from a molecule is the projection of Ewald-sphere cuts through the orbital distribution in k-space onto a plane representing the experimental geometry, e.g., the molecular plane in case of a molecule lying flat on a surface. The radius of the Ewald sphere is related to the kinetic energy of photoelectrons and thus to the photon energy: It is given by the latter minus the orbital energy with respect to the vacuum level. Note the common discrepancy between orbital energy as measured in experiment and calculated by theory. To account for this, we adapted the theoretical photon energy so that it results in the same kinetic energy for each experimental photon energy. However, for the sake of clarity and since the photon energy is the free-to-choose experimental parameter, momentum maps of identical kinetic energy are labeled according to the corresponding experimental photon energy.

Scanning tunneling microscopy (STM) measurements were performed at 77 K with a low-temperature STM apparatus attached to an UHV preparation chamber. Electrochemically etched tungsten tips were used.

The calculations for the molecular monolayer on three layers of Ag(001) and two layers of MgO(001) were carried out with the Vienna Ab initio Simulation Package^55−57^ plane wave code in the repeated-slab approach, where an interlayer vacuum of 18 Å was inserted alongside a dipole-correction in order to avoid spurious electric fields. We utilized the projector augmented wave method^58^ with an energy cutoff of 450 eV. The Brillouin zone was sampled with a Γ-centered mesh of 4 × 4 × 1 points, and we used a Gaussian-type smearing of the unoccupied states with 0.2 eV width.

In order to account for van der Waals (vdW) interactions, we have used two different approximations for the exchange-correlation treatment: PBE^59^ and the Tkatchenko–Scheffler method^49^ with iterative Hirshfeld-partitioning^60,61^ (PBE-TS) as well as the vdW-functional optb86b-vdw^50,51^ (vdW-DFT). The respective geometries were relaxed such that the maximum of the norm of the forces was lower than 0.005 eV/Å, while the 2 lowest layers of Ag(001) were held fixed (lattice constant: 4.16 Å). After geometry relaxation, the electronic structure was computed with the same parameters, albeit with a plane-wave cutoff of 500 eV and a Brillouin zone sampling of 8 × 8 × 3 points. Photoemission momentum maps for the full system as well as the molecular monolayer in the absorbed geometry were simulated in the plane-wave final-state approximation, as described, e.g., in ref (62). To calculate momentum maps of a gas-phase molecule, we used the molecular geometries from the aforementioned calculations and used the DFT module of the Gaussian orbitals based code NWChem^63^ to obtain the molecular orbitals. Here, the 6-31G** basis set and the B3LYP exchange–correlation functional^64,65^ were used. The momentum maps were then simulated from the real-space orbitals, as shown in ref (40).

## Supporting Information Available

LUMO and HOMO momentum maps as a function of energy. Photon energy dependence of simulated momentum maps. Dependence of the calculated PTCDA bend on the approach to treat van der Waals interaction and on the work function of the MgO(001)/Ag(001) substrate. Considerations beyond the plane-wave final-state approximation of isolated molecules. Methodology of TD-DFT calculations (PDF)
