# Supplementary material for: Large Distortion of Fused Aromatics on Dielectric Interlayers Quantified by Photoemission Orbital Tomography
Source: ACS Nano. 2022 Oct 14;16(10):17435–43. doi: 10.1021/acsnano.2c08631 (PMC9620409; doi:10.1021/acsnano.2c08631)
Supplement: Supplementary file 1 — nn2c08631_si_001.pdf [file nn2c08631_si_001.pdf]

Supporting information for

Large Distortion of Fused Aromatics on Dielectric Interlayers Quantified by Photoemission Orbital Tomography

Philipp Hurdax[1], Christian S. Kern[1], Thomas Georg Boné[1], Anja Haags[2,3,4], Michael Hollerer,[1] Larissa Egger[1], Xiaosheng Yang[2,3,4], Hans Kirschner[5], Alexander Gottwald[5], Mathias Richter[5], François C. Bocquet[2,3], Serguei Soubatch[2,3], Georg Koller[1], Frank Stefan Tautz[2,3,4], Martin Sterrer[1], Peter Puschnig[1] and Michael G. Ramsey[1]*

[1]Institute of Physics, University of Graz, NAWI Graz, Universitätsplatz 5, 8010 Graz, Austria.
[2]Peter Grünberg Institute (PGI-3), Forschungszentrum Jülich, 52425 Jülich, Germany.
[3]Jülich Aachen Research Alliance (JARA), Fundamentals of Future Information Technology, 52425 Jülich, Germany.
[4]Experimentalphysik IV A, RWTH Aachen University, 52074 Aachen, Germany.
[5]Physikalisch-Technische Bundesanstalt (PTB), 10587 Berlin, Germany.

* Corresponding author: E-Mail: Michael G. Ramsey – michael.ramsey@uni-graz.at

SI1. LUMO and HOMO momentum maps at different photon energies.

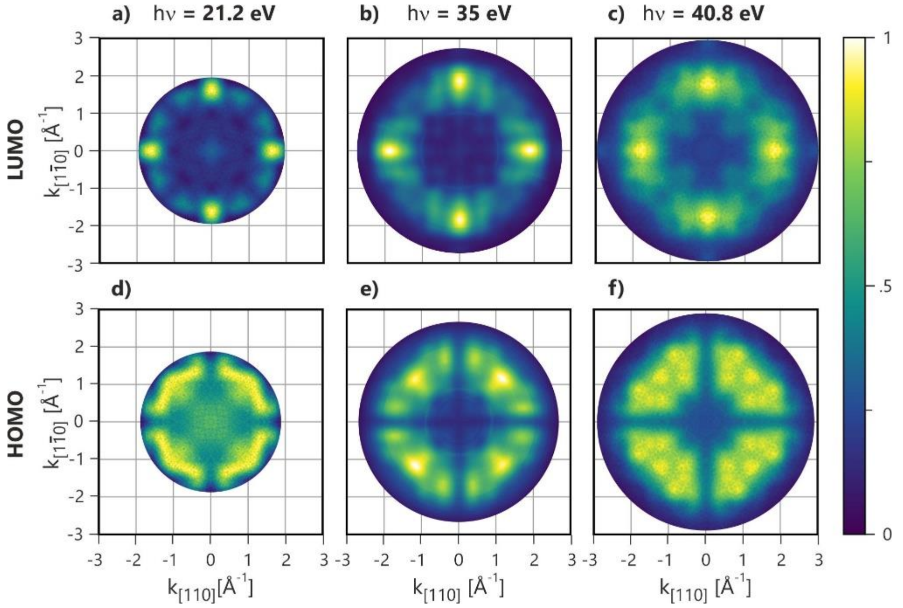

Fig. S1: *LUMO (a, b, c) and HOMO (d, e, f) maps taken at photon energies of (a, d) 21.2 eV, (b, e) 35 eV and (c, f) 40.8 eV. The symmetrized maps of a, c, d and f were measured with a Scienta Omicron NanoESCA system, while b and e were measured at the Metrological Light Source in Berlin with p polarized synchrotron radiation and a toroidal analyzer.*

SI2. Photon energy dependence of simulated LUMO and HOMO momentum maps of the bent molecule in the gas phase.

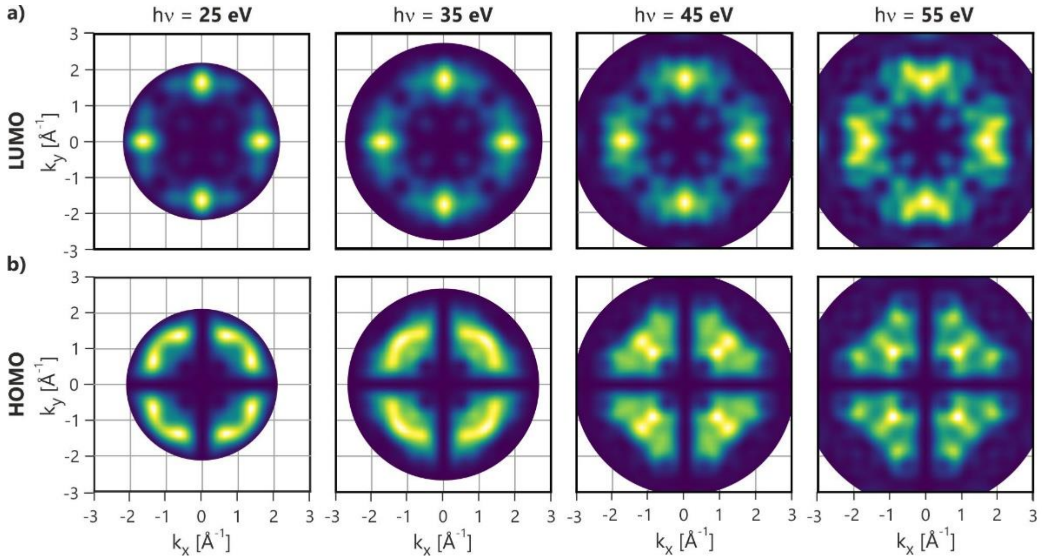

Fig. S2: *Displayed are* (a) *the LUMO and* (b) *the HOMO of PTCDA in the top and bottom rows, respectively. The molecular geometry with a C_{cent}-C_{carb} bend of* 0.67 Å *has been derived by DFT calculations on MgO(001)/Ag(001) using the PBE-TS approach.*

SI3. Dependence of the calculated PTCDA bend on the approach to treat the van der Waals interaction and on the work function of the MgO(001)/Ag(001) substrate

Table S1 summarizes the values of the PTCDA bend obtained with different approaches for treating the van-der-Waals forces and different densities of extra oxygen at the oxide-metal interface, which strongly affects the work function of the MgO(001)/Ag(001) substrate. The magnitude of the bend clearly depends on the type of calculation employed with the argueably more objective vdW DFT methodology resulting in a ~0.1 Å lower bend than that of the PBE-TS calculations. The bend decreasing for lower substrate work functions is related to the increase in charge transfer to the LUMO and concomitant increase in electrostatic attraction.

Table S1*: Calculated bends (C_{cent} – C_{carb}) in the carbon backbone of PTCDA on 2ML of MgO(001) on Ag(001) for different oxygen content at the MgO-Ag interface resulting in different work functions calculated with two different van der Waal correction schemes.*

Substrate	vdW DFT		PBE-TS	
	Φ_{sub} [eV]	C-Bend [Å]	Φ_{sub} [eV]	C-Bend [Å]
MgO / Ag(001)	2.96	0.59	2.84	0.67
MgO / ¼ ML O / Ag(001)	3.67	0.62	3.42	0.72
MgO / ½ ML O / Ag(001)	4.25	0.64	3.95	0.71
Ag(001)	4.23			

For the hypothetical bends of Figure 3 distortions beyond those predicted by the on surface calculations have been obtained by extrapolating the trajectories of the atomic positions within the molecule on the different work function substrates.

SI4. Considerations beyond the plane wave approximation of isolated molecules

To test if the discrepancy between experimental and simulated momentum maps might be explained by factors other than an altered shape of the molecular backbone, additional calculations have been carried out. Those comprised (i) a more accurate description of the final state with time-dependent density functional theory (TD-DFT), (ii) accounting for molecule-molecule interactions in the free-standing monolayer as well as (iii) including the substrate. The results are briefly discussed below.

i. Influence of the final-state approximation on the momentum maps

It has been suggested that the planarity of the molecule is a criterion for the validity of the plane-wave approximation [1]. Thus, it was first tested whether the approximation can properly capture the effect of the bend. This was achieved by simulating momentum maps for the isolated bent molecule with the time dependent DFT (TD-DFT) method, which essentially makes no assumptions of the final state (SI 5), and comparing these maps to the ones generated on the basis of the plane-wave approximation. As can be seen in **Fig. S3**, the k-map of the HOMO obtained from TD-DFT is very similar to that obtained with the plane-wave final state approximation. Thus, it is concluded that final-state effects cannot account for the observed discrepancies between theory and experiment, which therefore have been attributed to the geometry of the molecule being more distorted than predicted by the DFT calculations.

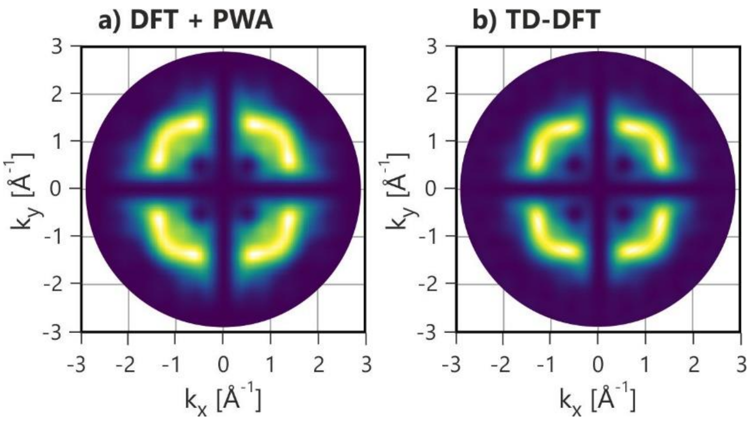

Fig. S3: Time-dependent DFT vs. the plane-wave approximation for bent PTCDA. *Momentum maps of the HOMO for the experimental photon energy of 40.8 eV obtained with* (a) *standard DFT and the plane-wave approximation and* (b) *the TD-DFT method. The maps are simulated to reflect the experimental setup of* ***Fig. S1f***.

ii. Influence of molecule-molecule interactions on the momentum maps

Secondly, the role of intermolecular interactions in the monolayer was evaluated by a DFT calculation of the free-standing monolayer with the atoms fixed to the positions found in calculations on the surface. The effect on the maps on the PTCDA LUMO and HOMO is shown in **Fig. S4** and **Fig. S5**, respectively. Here the simulations have the polarization factor included and have not been symmetrized to simulate the raw experimental momentum maps shown. The two figures compare the maps of the free-standing layer in the second row to maps of the isolated molecule in the first row and the experimental maps in the fourth row for three different photon energies (columns). While there is little change in the resulting momentum map of the LUMO, changes are slightly more pronounced in the map of the HOMO. However, including the intermolecular interactions results in no apparent improvement of the fit with the experimental maps.

iii. Influence of the substrate on the momentum maps

Thirdly, the influence of the substrate on the electronic structure was evaluated by comparing calculations of the molecule on the surface to the calculations of the isolated molecule. The former are shown for the LUMO and the HOMO in the third row of **Fig. S4** and **Fig. S5**, respectively. The comparison with the experimental maps in the fourth row of the figures shows that despite the use of damping in the final state to account for the surface sensitivity of POT [2], the contribution of the Ag substrate to the k-maps is significantly overestimated. Besides, the emission intensity of the HOMO is much more focused at the center of the emission feature compared to the free-standing molecular film, while the minor lobes of the LUMO adopt a more circular shape. This is surprising given that the frontier orbitals themselves are not involved in a chemical interaction with the substrate and even on metals, the influence of the substrate is small, as indicated by the close agreement between experimental maps and simulated maps of the molecule in the gas phase. However, accounting for the substrate does not lead to a clear improvement of the fit, and therefore a larger bend remains the most likely explanation for the differences between the theoretical and experimental maps.

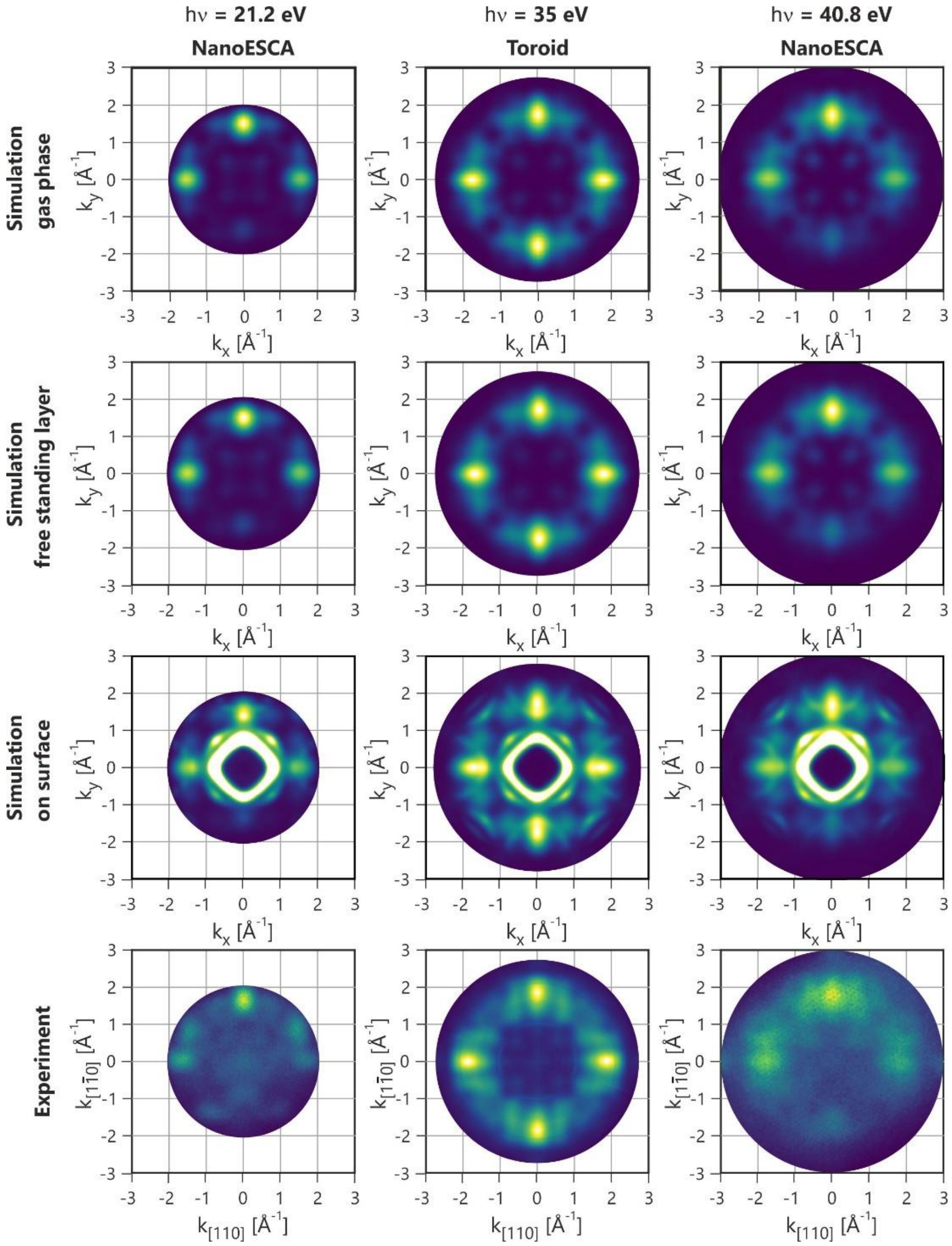

Fig. S4 Momentum maps of the PTCDA LUMO measured on MgO(001)/Ag(001) and simulated at three different photon energies. *1st row: Simulated momentum maps of the isolated bent molecule with its atomic positions frozen at the positions obtained by on-surface DFT calculations. 2nd row: Simulated momentum maps of a free-standing layer of molecules with their atomic positions frozen at the positions obtained by the on-surface DFT calculations. 3rd row: Simulated momentum maps including the substrate at the energy of the PTCDA HOMO. The surface sensitivity of UV photoemission has been accounted for by introducing an exponential damping with depth. The approximately circular feature close to the center of the maps originates from the Ag sp-band. 4th row: Momentum maps measured on MgO(001)/Ag(001). The measurement geometries, described in the methods section of the paper, were accounted for in the simulations in the first three rows of the figure. All simulations were carried out in the plane-wave approximation* [3].

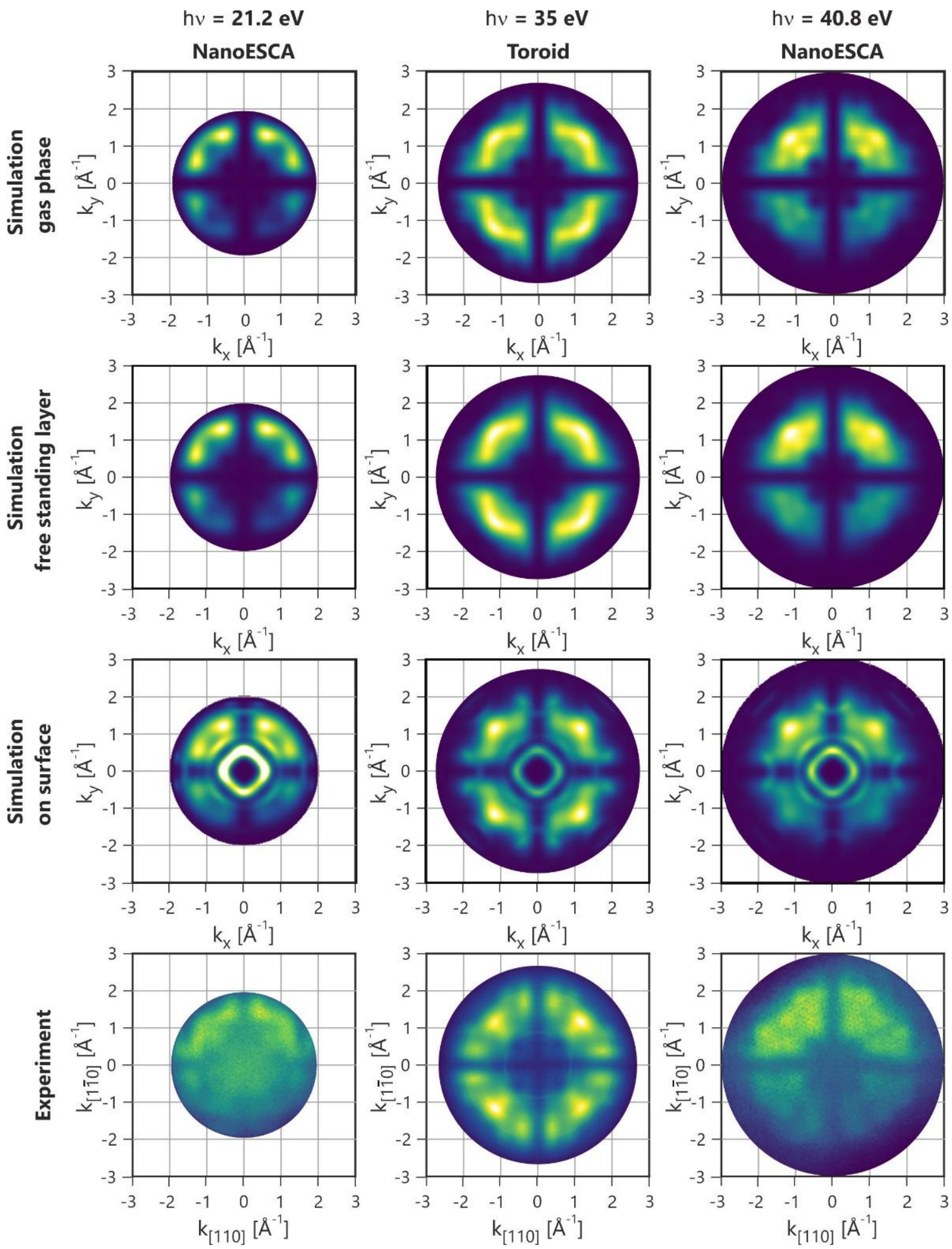

Fig. S5 Momentum maps of the PTCDA HOMO measured on MgO(001)/Ag(001) and simulated at three different photon energies. *As for the LUMO of Fig. S4.*

SI5. Methodology of the TD-DFT calculations

In time-dependent density functional theory calculations, the gas-phase molecule was subjected to a photon field and the flux of outgoing electron density was recorded over time [4] [5] with TD-DFT as implemented in the real-time real-space code OCTOPUS [6]. The electronic ground state was simulated in a spherical box (radius: 25 Å, grid spacing: 0.2 Å) and in the local density approximation [7] [8]. The system was then propagated for 10 fs, coupled to an electromagnetic field that would correspond to a laser with an intensity of $5{\cdot}10^8$ W/cm^2 at 40.8 eV photon energy. To eliminate non-adiabatic effects, the field was ramped up and down for 8 cycles each at the beginning and the end of the simulation. Throughout the simulation time, the outgoing photoelectron flux was recorded at half the simulation box radius, followed by a complex absorbing potential to avoid double-counting of reflected electrons [9].

References

[1] Bradshaw, A. M.; Woodruff, D. P. Molecular orbital tomography for adsorbed molecules: is a correct description of the final state really unimportant? *New J. Phys.* **2015**, *17*, 013033.

[2] Lüftner, D.; Weiß, S.; Yang, X.; Hurdax, P.; Feyer, V.; Gottwald, A.; Koller, G.; Soubatch, S.; Puschnig, P.; Ramsey, M. G.; Tautz, F. S. Understanding the photoemission distribution of strongly interacting two-dimensional overlayers. *Phys. Rev. B* **2017**, *96*, 125402.

[3] Brandstetter, D.; Yang, X.; Lüftner, D.; Tautz F. S.; Puschnig, P. kMap.py: A Python program for simulation and data analysis in photoemission tomography. *Comput. Phys. Commun.* **2021**, *263*, 107905.

[4] Wopperer, P.; De Giovannini U.; Rubio, A. Efficient and accurate modeling of electron photoemission in nanostructures with TDDFT. *Eur. Phys. J. B* **2017**, *90*, 1434-6036.

[5] De Giovannini, U.; Hübener H.; Rubio, A. A First-Principles Time-Dependent Density Functional Theory Framework for Spin and Time-Resolved Angular-Resolved Photoelectron Spectroscopy in Periodic Systems. *J. Chem. Theory Comput.* **2017**, 13, 1549-9618.

[6] Tancogne-Dejean N. et al. Octopus, a computational framework for exploring light-driven phenomena and quantum dynamics in extended and finite systems. *J. Chem. Phys.* **2020**, *152*, 124119.

[7] Dirac, P. A. M. Note on Exchange Phenomena in the Thomas Atom. *Math. Proc. Cambridge Philos. Soc.* **1930**, 26, 376.

[8] Perdew J. P.; Zunger, A. Self-interaction correction to density-functional approximations for many-electron systems. *Phys. Rev. B* **1981**, *23*, 5048-5079.

[9] De Giovannini, U.; Larsen A. H.; Rubio, A. Modeling electron dynamics coupled to continuum states in finite volumes with absorbing boundaries. *Eur. Phys. J. B* **2015***,* 88, 1434-6036.
